# The Study of Cell-Penetrating Peptides to Deliver dsRNA and siRNA by Feeding in the Desert Locust, *Schistocerca gregaria*

**DOI:** 10.3390/insects14070597

**Published:** 2023-07-01

**Authors:** Elise Vogel, Dulce Santos, Cissy Huygens, Paulien Peeters, Stijn Van den Brande, Niels Wynant, Jozef Vanden Broeck

**Affiliations:** 1 Research Group of Molecular Developmental Physiology and Signal Transduction, Division of Animal Physiology and Neurobiology, Department of Biology, KU Leuven, 3000 Leuven, Belgium; 2 Laboratory of Behavioral and Developmental Genetics, Department of Human Genetics, KU Leuven, 3000 Leuven, Belgium

**Keywords:** cell-penetrating peptide (CPP), RNA delivery system, double-stranded RNA (dsRNA), gene silencing, insect, naked RNA, oral RNAi, Orthoptera, pest control, small-interfering RNA (siRNA)

## Abstract

**Simple Summary:**

This study focuses on the desert locust, a critical insect pest species for agriculture. We aimed at investigating how to induce RNA interference by feeding in this species. RNA interference is a gene-silencing mechanism that promises to contribute to pest control strategies. We studied cell-penetrating peptides (CPPs) as potential dsRNA/siRNA delivery systems. We found CPPs that can complex with dsRNA and siRNAs, as well as protect them from the degradation of midgut enzymes. In addition, we report that intra-hemocoelic injection of naked siRNAs does not trigger a gene-silencing response in the desert locust but, for siRNAs complexed with one of the investigated CPPs, it does. Although we could not find a suitable CPP to induce RNAi by feeding in the locust, our results stimulate future research on this topic. In addition, our findings contribute to the understanding of the RNA interference response and its complexity in insects as well as emphasizing the importance of research in living insects when it comes to dsRNA/siRNA oral delivery systems.

**Abstract:**

RNA(i) interference is a gene silencing mechanism triggered by double-stranded (ds)RNA, which promises to contribute to species-specific insect pest control strategies. The first step toward the application of RNAi as an insecticide is to enable efficient gene silencing upon dsRNA oral delivery. The desert locust, *Schistocerca gregaria* is a devastating agricultural pest. While this species is responsive to dsRNA delivered by intra-hemocoelic injection, it is refractory to orally delivered dsRNA. In this study, we evaluated the capacity of five cell-penetrating peptides (CPPs) to bind long dsRNA and protect it from the locust midgut environment. We then selected the CPP EB1 for further in vivo studies. EB1:dsRNA complexes failed to induce RNAi by feeding. Interestingly, we observed that intra-hemocoelic injection of small-interfering (si)RNAs does not result in a silencing response, but that this response can be obtained by injecting EB1:siRNA complexes. EB1 also protected siRNAs from midgut degradation activity. However, EB1:siRNA complexes failed as well in triggering RNAi when fed. Our findings highlight the complexity of the dsRNA/siRNA-triggered RNAi in this species and emphasize the multifactorial nature of the RNAi response in insects. Our study also stresses the importance of in vivo studies when it comes to dsRNA/siRNA delivery systems.

## 1. Introduction

RNA interference (RNAi) is a post-transcriptional gene silencing mechanism triggered by dsRNA. In short, dsRNA molecules are recognized in the cytoplasm by the RNase III enzyme Dicer2 and processed into small interfering RNAs (siRNAs, 18–23 bp long). These small RNA duplexes are incorporated into an RNA-induced silencing complex (RISC), which is then directed to a messenger RNA target via Watson–Crick base pairing. Subsequently, the effector protein of RISC, namely Argonaute2, acts to inhibit or degrade the specific transcript, resulting in reduced gene expression. While naturally activated as an antiviral response, this mechanism can be triggered by artificially delivered gene-specific long dsRNA, leading to targeted endogenous gene silencing. This is called RNAi technology and is widely used in functional genomics research for studying loss-of-function phenotypes. In addition, RNAi technology constitutes a promising technique for pest control as it can contribute to the development of novel, highly specific insecticides, based on the silencing of strategically selected genes [1].

When discussing the potential of RNAi for biotechnological applications in insects, it is relevant to consider environmental RNAi. This refers to the initial uptake of dsRNA molecules from an extracellular environment such as the midgut lumen or the hemolymph, followed by the consequential RNAi-based gene silencing in these cells [2]. While it remains unclear how these mechanisms take place in different insect species, there is the consensus that the efficiency of environmental RNAi depends on two main factors: (i) the stability of the dsRNA molecules in the extracellular environment prior to uptake; (ii) and the existence of an efficient dsRNA cellular uptake system. In this regard, the route by which the dsRNA is administered is also critical. In some species, injection of dsRNA in the hemocoel can induce RNAi, while ingestion does not. An interesting example is the desert locust, *Schistocerca gregaria*, which is an agricultural pest of serious concern, constantly monitored by FAO—Food and Agriculture Organization [3]. This animal displays a very robust RNAi response upon injection of dsRNA in the hemolymph. However, this species does not respond to dsRNA delivered by feeding [4,5], compromising the possibility of pest control through feeding-based RNAi. A possible solution to this problem is presented by packaging dsRNA in such a way that it is protected against degradation and that the uptake is facilitated—a dsRNA delivery system. Although several types of dsRNA delivery systems have been proposed in insects, including microorganisms [6,7,8,9,10,11,12,13,14,15,16,17], nanoparticles [18,19,20,21,22], and liposomes [23,24,25,26], cell-penetrating peptides (CPPs) are understudied in this context.

CPPs constitute an extremely diverse class of carrier molecules, mainly characterized by their small size and ability to facilitate cellular uptake of a large range of bio-active macromolecules [27]. CPPs are short-chain peptides that usually consist of 10–30 amino acids with a high prevalence of basic residues, such as lysine and arginine [28]. While more than one thousand CPPs have been registered or patented for applications ranging from tumor therapy to protein delivery in mammalian cells [29], their use in insects remains to be explored. Cermenati et al. (2011) found that the CPP TAT could improve the uptake of enhanced Green Fluorescent Protein in cultured columnar midgut cells of the domestic silkworm, *Bombyx mori* [30]. Moreover, three arginine-rich CPPs, namely HR9, SR9, and PR9, were reported to facilitate the uptake of plasmid DNA in Sf9 cells, a cell line derived from ovarian tissue of the fall armyworm, *Spodoptera frugiperda* [31]. Additionally, feeding a fusion protein of TAT and diapause hormone to larvae of the cotton bollworm, *Helicoverpa armigera*, resulted in stunted larval growth [32]. Most relevantly, research has shown that a PTD-dsRBD fusogenic CPP could act as a vehicle for the oral delivery of dsRNA, increasing RNAi efficiency in *Anthonomus grandis* by protecting the dsRNA against degradation and facilitating its cellular uptake [33]. These examples illustrate the promising application potential of CPP-mediated cellular uptake in insects. Moreover, their high efficiency, versatility, and low cytotoxicity make these peptide carriers interesting candidates for further application as a delivery system.

In this study we investigated the potential of CPPs to contribute to RNAi by feeding in the desert locust, *S. gregaria*. We started by selecting five CPPs based on their potential ability to bind RNA duplexes non-covalently through electrostatic interaction, as well as to facilitate endosomal escape [34,35,36,37,38,39,40,41,42,43,44]. In total, five CPPs were tested: two endosomolytic CPPs, namely the penetratin analog EB1 and C6M1; two fusogenic CPPs, namely HA2-penetratin and HA2-TAT; and the polyarginine peptide POA (Table 1). These CPPs were tested for their ability to form complexes with long dsRNA, as well as for their capacity to protect these molecules from degradation in the *S. gregaria* midgut environment. We then proceeded to test if EB1 could function as a dsRNA delivery system to trigger RNAi by feeding in the desert locust. Under the tested conditions, feeding of EB1:dsRNA complexes failed to induce an RNAi response. We have also observed that, in contrast to long dsRNA, siRNAs do not induce RNAi in *S. gregaria* upon injection, but when EB1:siRNA complexes were injected, a significant knockdown was obtained. EB1 also protected siRNAs from midgut degradation activity. Nevertheless, EB1:siRNA complexes were still unable to trigger RNAi by feeding under the assayed conditions in *S. gregaria*.

## 2. Materials and Methods

### 2.1. Rearing of the Desert Locust, S. gregaria

*S. gregaria* was reared under crowded conditions (>200 locusts per cage) at a controlled temperature of 30 °C (±1 °C), a photoperiod cycle of 14/10 h light/dark, and an ambient relative humidity between 40% and 60%. These insects were fed ad libitum with a mix of cabbage and corn leaves, supplemented with rolled oats. After mating, adult female insects were allowed to deposit their eggs in pots containing a moistened soil mixture consisting of 75% turf with 25% sand. Egg pots were collected weekly and placed in a fresh cage where the first nymphal instar locusts were allowed to hatch.

### 2.2. CPPs

#### 2.2.1. CPP Synthesis and Purification

All crude peptides were commercially synthesized by Synpeptide. The amino acid sequences for these peptides are represented in Table 1. Further purification of the crude peptides was achieved by high-performance liquid chromatography (HPLC) using a SunFire Prep C18 5 μM column (10 × 250 mm). The mobile phase consisted of acetonitrile (10%) supplemented with trifluoroacetic acid (TFA, 0.1%) to improve the separation of the sample components. Mass spectrometry (MS/MS) was then used to determine which fraction contained the correct peptide sequence. The organic solvent was removed by evaporation under vacuum using the SpeedVac concentrator SVC 200 H/100 H (Savant). Peptides were re-dissolved in phosphate-buffered saline (PBS; pH 7.4) and transferred to LoBind microcentrifuge tubes.

#### 2.2.2. CPP Quantification

The peptide concentration was determined by means of the bicinchoninic acid (BCA) protein assay. This assay utilizes two different reagents, which will henceforth be referred to as reagents A and B. Reagent A consists of 0.1% bicinchoninic acid, 2% NaCO_3_, 0.16% Na_2_C_4_H_4_O_6_, 0.1 M NaOH. This solution was adjusted to pH 11.25 using NaOH. Reagent B is created by dissolving 0.4% CuSO_4_.5H_2_O in 50 mL of distilled water. Reagents A and B were mixed in a 50:1 ratio. To determine CPP concentration, samples were compared to a Bovine Serum Albumin (BSA) standard series. OD was measured at a wavelength of 563 nm using the NanoPhotometer^®^ N60 spectrophotometer (Implen).

### 2.3. Synthesis of Long dsRNA

*Luciferase* dsRNA (ds*Luc*; 1054 bp) was commercially synthesized by Genolution and provided by Syngenta (Ghent, Belgium). *S. gregaria alpha-tubulin 1a* dsRNA (ds*Tub*; 545 bp) was created using the MEGAscript^®^ RNAi kit (Ambion) according to the manufacturer’s protocol. This kit utilizes a T7 polymerase to transcribe RNA from a DNA template containing a T7 promoter site. Therefore, T7 promoter sites were added to the template DNA through a PCR reaction using the RED-Taq^®^ ReadyMix (Merck) and the primers listed in Table 2. Primers for ds*Tub* were previously described by Wynant et al. (2012) [4]. The amplification product was verified using agarose gel electrophoresis and visualized using UV and a GelredTM Nucleic Acid Gel Stain (Biotium). dsRNA concentration and purity were determined using the NanoPhotometer^®^ N60 spectrophotometer (Implen). Additionally, dsRNA integrity was verified using agarose gel electrophoresis.

### 2.4. Synthesis of Fluorescent dsRNA

Fluorescent dsRNA was produced by labeling long dsRNA constructs with Cy3 using the Silencer^®^siRNA labeling kit (Ambion) according to the manufacturer’s protocol. dsRNA molecules were separated from unbound Cy3 and other contaminating reagents through Ethanol (EtOH) precipitation. dsRNA labeling was confirmed by visualizing and assessing fluorescence using an EthanDIGE imager (GE Healthcare), after separation through agarose gel electrophoresis. dsRNA concentration and labeling efficiency were finally determined using the NanoPhotometer^®^ N60 spectrophotometer (Implen).

### 2.5. Synthesis of (Fluorescent) siRNAs

siRNAs of (fluorescently labeled) dsRNA (ds*Tub* and ds*Luc*), henceforth named ds*Tub*-siRNA and ds*Luc*-siRNA, were created using the ShortCut RNAi Kit (New England Biolabs) according to the manufacturer’s instructions. siRNAs were purified from contaminating reagents through EtOH precipitation. siRNA pellets were dissolved in Milli-Q water (MQ; Millipore) and sonicated with the Digital Sonifier^®^ SLP (Branson) to ensure that all siRNAs were in solution. siRNA concentration and purity were verified using the NanoPhotometer^®^ N60 spectrophotometer (Implen). Agarose gel electrophoresis was used to check if the dsRNA template had been fully digested.

### 2.6. Formation of CPP:dsRNA and CPP:siRNA Complexes

CPPs were complexed with the long dsRNA fragments ds*Tub* and ds*Luc*, as well as with the corresponding siRNAs (ds*Tub*-siRNA and ds*Luc*-siRNA). Complexation was realized by an electrostatic binding interaction between the negatively charged RNA duplex molecules and the positively charged peptides. For this purpose, dsRNA or siRNAs were mixed with each CPP and incubated on ice for 30 min. This reaction was performed at different w:w ratios to evaluate the amount of CPP needed for optimal complexation and protection. Tested ratios were 1:1, 2:1, 5:1, and 10:1 for the CPP:dsRNA complexes, and 1:1, 2:1, and 5:1 for the CPP:siRNA complexes. Converted molar ratios of these complexes can be found in Table 3 for complexes formed with ds*Tub* and ds*Luc*-siRNA. Agarose gel electrophoresis was used to check the success of the complexation, as previously described [34,44,48,49].

### 2.7. Assessment of Complex Stability in an S. gregaria Ex Vivo Gut Environment

#### 2.7.1. Collection of Midgut Enzyme Solution

Midgut biologically active solution was collected from dissected midguts of adult *S. gregaria* in an isotonic *Sg*-Ringer solution (8.766 g/L NaCl, 0.188 g/L CaCl2, 0.746 g/L KCl, 0.407 g/L MgCl2, 0.336 g/L NaHCO_3_, 30.807 g/L sucrose, and 1.892 g/L trehalose; pH 7.2). Total enzyme content from the midguts of five separate insects was collected in 500 μL of *Sg*-Ringer. To minimize debris from (un)digested food, insects were starved overnight before taking these samples. The remaining debris in the midgut enzyme solution (MgES) samples was pelleted by centrifugation at 3000× *g* for 10 min (4 °C). Hereafter, the cleared MgES was transferred to new Eppendorf tubes and stored at −20 °C for up to a month.

#### 2.7.2. Ex Vivo Degradation Assay

**Figure 1:**
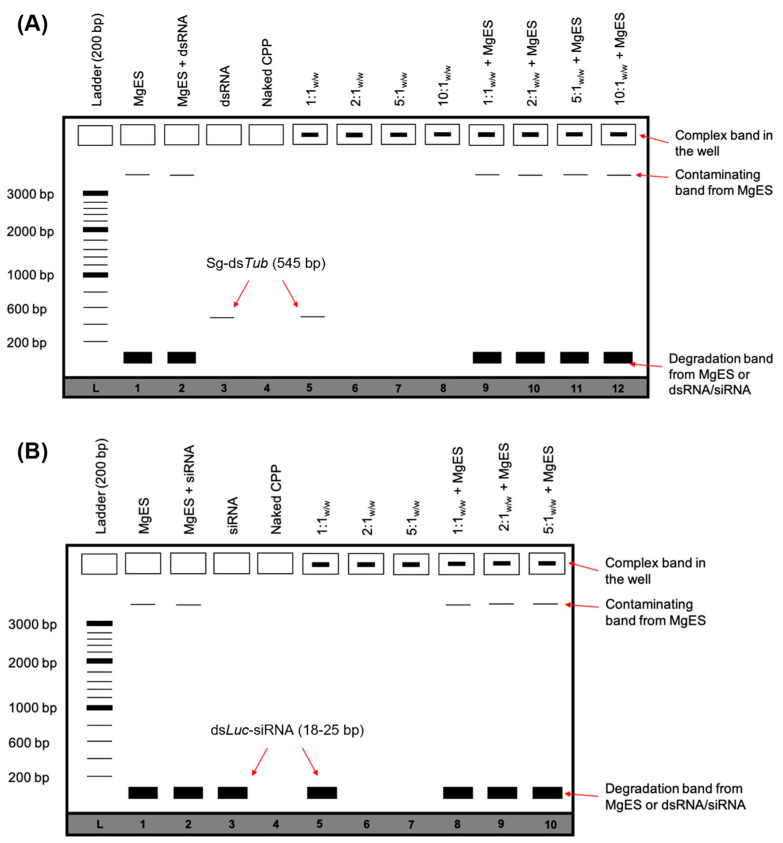
Schematic representation of the expected agarose gel electrophoresis bands after performing an ex vivo degradation assay for CPP:dsRNA (**A**) or CPP:siRNA (**B**) complexes. This figure is intended as a guide to Section 3. When dsRNA/siRNA is bound by the CPP, the resulting complex bands are visualized in the well. If the complex is degraded during the assay, this band disappears. However, if the CPP protects dsRNA/siRNA from degradation, this band remains visible even after complexes are incubated in MgES. Incubated samples may contain additional bands from the utilized MgES. These contaminating bands appear as background fluorescence and represent residual nucleic acid material from plant or endogenous source. Complexes are formed with ds*Luc* (545 bp) or ds*Luc*-siRNA (18–25 bp). If binding to the CPP is inefficient, a residual dsRNA/siRNA band can be visualized in the lanes containing the control complexes. Results are always viewed in reference to a 200 bp ladder (L).

The ability of CPP:dsRNA and CPP:siRNA complexes to withstand degradation in the aggressive environment of the gut was tested in an ex vivo degradation assay using MgES. For this, complexes were created as described in Section 2.6 at w:w ratios of CPP:dsRNA 1:1, 2:1, 5:1, and 10:1; and of CPP:siRNA 1:1, 2:1, 5:1. All complexes were created using 50 ng/µL of ds*Tub* or ds*Luc*-derived siRNAs (ds*Luc*-siRNA), while the amount of CPP was adjusted according to the intended ratio. 20 μL of complex solution for each individual CPP was then added to an equal amount of MgES. Degradation was allowed to take place for a total duration of 2 h: samples were taken at 5 min, 30 min, 60 min, and 120 min after the incubation reaction was initiated. At each time point, 5 μL was removed from the degradation reaction to assess the integrity of the incubated complex through a 1% agarose gel electrophoresis, in line with previously described approaches [33,46]. In order to track the samples, 6× DNA loading dye (Thermo Fisher Scientific, Merelbeke, Belgium) was added. As a control, naked ds*Tub* or ds*Luc*-siRNA were similarly incubated in MgES. Degradation of control dsRNA or siRNA was evaluated at the same time points. Additionally, MgES, the naked CPP, the naked ds*Tub* or ds*Luc*-siRNA, as well as the native complexes, were taken as controls. All images were created using the ProXima 2500 imager (Isogen Life Science, Utrecht, The Netherlands). dsRNA was visualized under UV light using a GelredTM Nucleic Acid Gel Stain (Biotium). As a guide to the results, a schematic overview of the expected agarose gel electrophoresis bands after ex vivo degradation of the complexes was included (Figure 1).

### 2.8. Visualization of EB1:dsRNA and EB1:siRNA Complexes

CPP complexes were visualized using the SteREO discovery v8 stereoscopic microscope (ZEISS). For this, 50 ng/µL fluorescently labeled ds*Luc* or ds*Luc*-siRNA were complexed with EB1 in a 5:1_w:w_ ratio. Complexes were dissolved in MQ before distribution onto a carrier glass. Per condition, technical replicates from one independent sample were analyzed. In order to visualize the Cy3 fluorescent marker, complexes were excited at 532 nm. Pictures were created using the AxioCam HRm camera (ZEISS).

### 2.9. RNAi Assays

#### 2.9.1. Feeding Assays

Feeding assays were performed using EB1:dsRNA and EB1:siRNAs complexes (for *Tub*; and *Luc*, as negative control). Each complex was dissolved in a total volume of 10 μL using PBS (pH 7.4). Complexes were then force-fed to adult *S. gregaria* by pipetting them directly into the insect’s mouth. Treated insects were immediately placed on food to allow normal feeding. As a control, insects were also fed with PBS or the naked EB1. The insects were treated daily for a period of 5 or 8 consecutive days, after which the midgut and brain were dissected.

Long dsRNA. EB1:dsRNA complexes were created for ds*Tub*, and ds*Luc* as a negative control. Complexes were created using 10 ng, 100 ng, or 300 ng of the respective dsRNA constructs. The quantity of EB1 (respectively, 50 ng, 500 ng, or 1500 ng) was adjusted to create complexes of a 5:1_w:w_ ratio (EB1:dsRNA).

siRNA. EB1:siRNA complexes were created for ds*Tub*-derived siRNAs (ds*Tub*-siRNA), and ds*Luc*-derived siRNAs as a negative control (ds*Luc*-siRNA). Complexes were created using 250 ng or 1000 ng siRNAs. Based on this, the amount of EB1 (1250 ng or 5000 ng, respectively) was adjusted to a ratio of 5:1_w:w_ (EB1:siRNA).

#### 2.9.2. Injection Assays

Naked dsRNA or siRNA (500 ng), as well as EB1:siRNA complexes, were injected into the body cavity of adult locusts. Injected complexes were always formed at a ratio of 5:1_w:w_ (EB1:siRNA). Complexes were formed using 2500 ng of EB1 and 500 ng ds*Tub*-derived siRNAs (ds*Tub*-siRNA) or ds*Luc*-derived siRNAs (ds*Luc*-siRNA) as a control. The siRNA/dsRNA and complexes were dissolved in *Sg*-Ringer before injection. Adult insects were then injected with 4 μL of the solutions. A boost injection was performed after 3 days. Insect midguts were dissected on day 6 of the experiment.

### 2.10. Tissue Collection

Midguts were removed from the insects and thoroughly cleaned; all food debris and Malpighian tubules were removed. Brains were dissected under a binocular microscope. All tissues were rinsed in *Sg*-Ringer, collected in MagNa Lyser Green Beads tubes, and snap-frozen in liquid nitrogen to prevent tissue degradation. Pools containing tissues of three individual insects were created for each experimental condition. All collected tissues were stored at −80 °C until further use.

### 2.11. RNA Extraction and cDNA Synthesis

Tissues collected in MagNa Lyser Green Beads tubes were first disrupted and homogenized in 1 mL QIAzol lysis reagent (QIAGEN^®^, Hilden, Germany) using the MagNa Lyser instrument (6500 rpm; 30 s). RNA was extracted from this homogenate using the RNeasy Lipid Tissue Mini Kit (QIAGEN^®^) according to the manufacturer’s protocol. A DNase digestion step (RNase-free DNase set, QIAGEN^®^) was included to remove contaminating genomic DNA from the purified RNA. The purity and concentration of the extracted RNA samples were measured using the NanoPhotometer^®^ N60 spectrophotometer (Implen). cDNA was then created for all RNA samples using the PrimeScript™ RT reagent kit (Takara^®^) according to the manufacturer’s protocol. This kit utilizes a reverse transcriptase, as well as random hexamers and oligo(dT) as primers, to create cDNA from RNA templates. The final reaction product was diluted 10-fold in MQ (Millipore). Samples were stored at −20 °C until further use.

### 2.12. Quantitative Real-Time PCR (qRT-PCR)

Relative transcript levels of *alpha tubulin 1a* (*Tub*) were measured through quantitative real-time PCR (qRT-PCR). All reactions were performed in duplicate in a 96-well plate using the StepOne Plus™ Real-Time PCR System (ABI Prism, Applied Biosystems, Waltham, MA, USA). Each reaction contained 0.75 µL transcript-specific forward (Fw) and reverse (Rv) primers (10 μM), 2.25 μL MQ, and 7.5 µL Fast SYBR^®^ Green Master Mix (Applied Biosystems) and 3.75 µL cDNA. Primers for the amplification of *Tub* were selected according to Van Hiel et al. (2009) [50]. New primers were designed using the Primer3Plus (available online) as well as the OligoAnalyzer Tool (IDT) to predict the formation of secondary structures and primer dimers. The efficiency of the selected primers was validated by performing qRT-PCR on a standard dilution series to determine primer annealing efficiency, followed by a dissociation protocol to exclude the formation of primer dimers. All selected primers returned a single melting peak. The amplification product was further analyzed using agarose gel electrophoresis and the bands from this gel were excised. The DNA was purified using the GenElute™ Gel Extraction kit, according to the manufacturer’s protocol (Sigma Aldrich), and cloned in the pCR™4-TOPO^®^ TA vector for sequencing (TOPO^®^ TA cloning kit for sequencing, Invitrogen) to confirm amplification specificity. All selected primers showed a single amplification product after agarose gel electrophoresis. Primer sequences are represented in Table 4.

Relative transcript levels of the target gene *Tub* were calculated according to the ΔΔCt method [51]. Two housekeeping genes were used, which were chosen for their relatively stable expression. These genes were selected from a pool of previously established candidate reference genes [50]. Transcript expression stability of these genes was evaluated in all tested conditions using the geNorm software and the following housekeeping genes were selected: *elongation factor 1a* (*EF1a*), *glyceraldehyde phosphate dehydrogenase* (*GAPDH*), and *ubiquitin* (*Ubi*). Target gene expression in the brain was normalized against expression of both *EF1a* and *Ubi*. For midgut, *GAPDH* and *EF1a* were used as reference genes. When all samples from one experiment could not be measured in the same 96-well plate, an inter-plate calibrator sample was used to calibrate for inter-plate run differences. Additionally, a no-template control (NTC) was added to each plate to test for primer-dimer formation or any other contaminants that could give rise to false-positive results. Statistical analysis was performed using GraphPad Prism 6 for Windows version 6.01 (GraphPad Software Inc., San Diego, CA, USA).

## 3. Results

### 3.1. CPP:dsRNA Complexation and Stability in Ex Vivo Locust Midgut Environment

**Figure 2:**
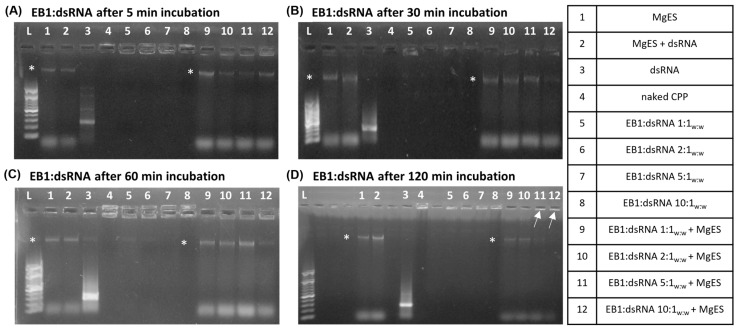
Ex vivo degradation assay of EB1:dsRNA in MgES. Complexes were incubated for 5 min (**A**), 30 min (**B**), 60 min (**C**), and 120 min (**D**) to determine stability of EB1:dsRNA in an *S. gregaria* midgut environment. A 200 bp ladder was used (L). As a control, incubated complexes were compared to pure MgES (1), naked dsRNA incubated in MgES (2), naked dsRNA (3), EB1 (4), and the native EB1:dsRNA complexes at ratios of 1:1_w:w_ (5), 2:1_w:w_ (6), 5:1_w:w_ (7) and 10:1_w:w_ (8). Complexes were incubated in MgES at ratios of 1:1_w:w_ (9), 2:1_w:w_ (10), 5:1_w:w_ (11), and 10:1_w:w_ (12). White arrows indicate complex bands after 120 min incubation in MgES. Furthermore, some background fluorescence was observed in all samples containing MgES: an asterisk (*) indicates the presence of a contaminating band representing residual nucleic acid material from a plant or endogenous source.

**Figure 3:**
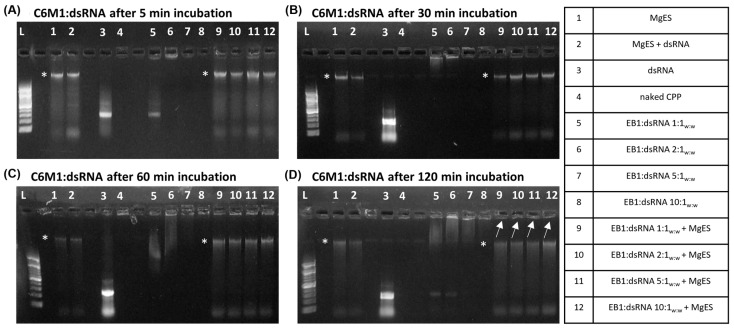
Ex vivo degradation assay of C6M1:dsRNA in MgES. Complexes were incubated for 5 min (**A**), 30 min (**B**), 60 min (**C**), and 120 min (**D**) to determine stability of C6M1:dsRNA in an *S. gregaria* midgut environment. A 200 bp ladder was used (L). As a control, incubated complexes were compared to pure MgES (1), naked dsRNA incubated in MgES (2), naked dsRNA (3), C6M1 (4), and the native C6M1:dsRNA complexes at ratios of 1:1_w;w_ (5), 2:1_w:w_ (6), 5:1_w:w_ (7) and 10:1_w:w_ (8). Complexes were incubated in MgES at ratios of 1:1_w:w_ (9), 2:1_w:w_ (10), 5:1_w:w_ (11), and 10:1_w:w_ (12). White arrows indicate complex bands after 120 min incubation in MgES. Furthermore, some background fluorescence was observed in all samples containing MgES: an asterisk (*) indicates the presence of a contaminating band representing residual nucleic acid material from a plant or endogenous source.

**Figure 4:**
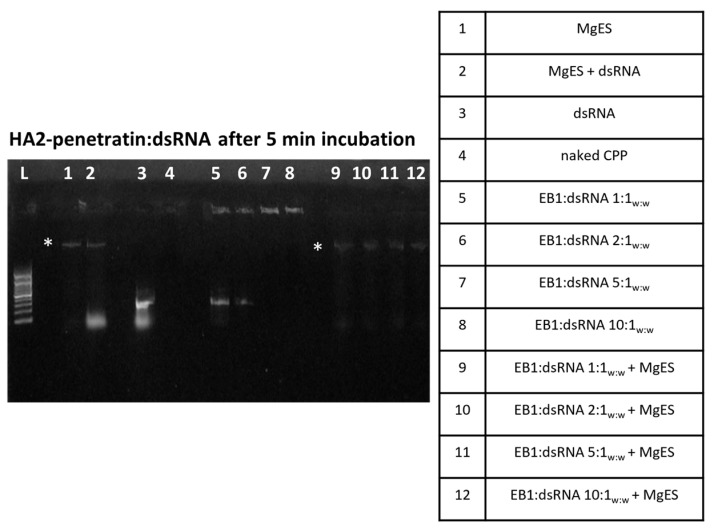
Ex vivo degradation assay of HA2-penetratin:dsRNA in MgES. Complexes were incubated for 5 min to determine stability of HA2-penetratin:dsRNA in an *S. gregaria* midgut environment. A 200 bp ladder was used (L). As a control, incubated complexes were compared to pure MgES (1), naked dsRNA incubated in MgES (2), naked dsRNA (3), HA2-penetratin (4), and the native HA2-penetratin:dsRNA complexes at ratios of 1:1_w:w_ (5), 2:1_w:w_ (6), 5:1_w:w_ (7), and 10:1_w:w_ (8). Complexes were incubated in MgES at ratios of 1:1_w:w_ (9), 2:1_w:w_ (10), 5:1_w:w_ (11), and 10:1_w:w_ (12). Some background fluorescence was observed in all samples containing MgES: an asterisk (*) indicates the presence of a contaminating band representing residual nucleic acid material from a plant or endogenous source.

**Figure 5:**
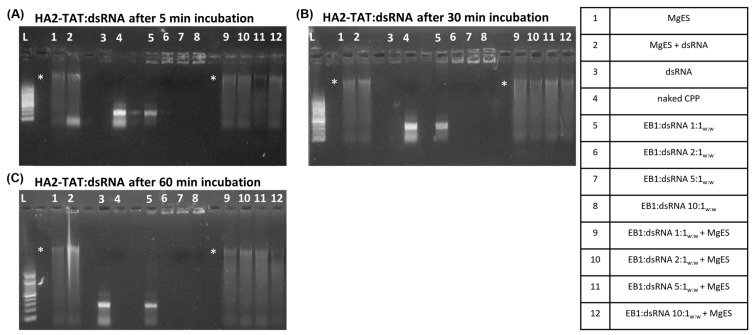
Ex vivo degradation assay of HA2-TAT:dsRNA in MgES. Complexes were incubated for 5 min (**A**), 30 min (**B**), 60 min (**C**) to determine stability of HA2-TAT:dsRNA in an *S. gregaria* midgut environment. A 200 bp ladder was used (L). As a control, incubated complexes were compared to pure MgES (1), naked dsRNA incubated in MgES (2), naked dsRNA (3), HA2-TAT (4), and the native HA2-TAT:dsRNA complexes at ratios of 1:1_w:w_ (5), 2:1_w:w_ (6), 5:1_w:w_ (7), and 10:1_w:w_ (8). Complexes were incubated in MgES at ratios of 1:1_w:w_ (9), 2:1_w:w_ (10), 5:1_w:w_ (11), and 10:1_w:w_ (12). Some background fluorescence was observed in all samples containing MgES: an asterisk (*) indicates the presence of a contaminating band representing residual nucleic acid material from a plant or endogenous source.

**Figure 6:**
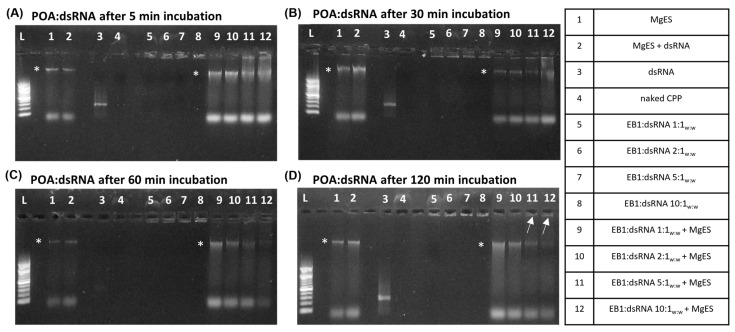
Ex vivo degradation assay of POA:dsRNA in MgES. Complexes were incubated for 5 min (**A**), 30 min (**B**), 60 min (**C**), and 120 min (**D**) to determine stability of POA:dsRNA in an *S. gregaria* midgut environment. A 200 bp ladder was used (L). As a control, incubated complexes were compared to pure MgES (1), naked dsRNA incubated in MgES (2), naked dsRNA (3), POA (4), and the native POA:dsRNA complexes at ratios of 1:1_w:w_ (5), 2:1_w:w_ (6), 5:1_w:w_ (7), and 10:1_w:w_ (8). Complexes were incubated in MgES at ratios of 1:1_w:w_ (9), 2:1_w:w_ (10), 5:1_w:w_ (11), and 10:1_w:w_ (12). White arrows indicate complex bands after 120 min incubation in MgES. Furthermore, some background fluorescence was observed in all samples containing MgES: an asterisk (*) indicates the presence of a contaminating band representing residual nucleic acid material from a plant or endogenous source.

We started by testing five CPPs (Table 1) for capacity to complex with dsRNA and protect it against the aggressive environment of *S. gregaria* gut. In parallel, we performed an ex vivo degradation assay, in which we tested the ability of each CPP to protect long dsRNA in MgES, an enzyme solution collected from the midgut of *S. gregaria*. A schematic guide of the experimental results is depicted in Figure 1. As expected from previous work [5], naked ds*Tub* was fully degraded after a 5 min incubation in MgES in every test (Figure 2, Figure 3, Figure 4, Figure 5 and Figure 6, well 2); background fluorescence was present in MgES, as indicated by the presence of multiple bands in the pure MgES control (Figure 2, Figure 3, Figure 4, Figure 5 and Figure 6, well 1). Accordingly, the same bands appeared during all MgES incubation reactions (Figure 2, Figure 3, Figure 4, Figure 5 and Figure 6, wells 2 and 9–12). This is indicated with an asterisk (*) in the figures. Each CPP-specific result is displayed separately, in continuation.

EB1 was able to completely bind the dsRNA starting at a ratio of 1:1_w:w_ (CPP:dsRNA), as evidenced by the complex band visualized in the well for all tested ratios (Figure 2A–D, well 5–8). For 1:1_w:w_ complexes, a band was still visible after 5 min of incubation in MgES, while this was not the case after 30 min, indicating that some degradation took place during this period (Figure 2A,B, well 9). Complexes 2:1_w:w_ remained stable for up to 60 min in MgES but were almost fully degraded at 120 min, with only a very light band remaining (Figure 2A–D, well 10). A clear complex band was present in the well for EB1:dsRNA complexes formed at a ratio of 5:1_w:w_ and 10:1_w:w_ for the entire duration of the experiment (Figure 2A–D, well 11 and 12), indicating high stability of these complexes.

C6M1 showed incomplete binding to long dsRNA at a ratio of 1:1_w:w_ and 2:1_w:w_ (C6M1:dsRNA), as evidenced by the presence of a weak band at the height of the unbound control dsRNA (Figure 3A,C,D, well 5 and 6). Nevertheless, partial binding still seemed to have taken place as a complex band also appeared in the well. Complexation of C6M1 with dsRNA was more successful at the higher w:w ratios of 5:1_w:w_ and 10:1_w:w_, at which the CPP completely bound all dsRNA (Figure 3A–D, well 7 and 8). The complex showed remarkable stability in MgES. Although some degradation occurred, as is evidenced by the reduced intensity of the incubated complex bands in comparison to those representing the control complexes, a complex band remained visible in the well for all incubated ratios for the full duration of the experiment (Figure 3A–D, well 9–12).

HA2-penetratin showed a relatively low dsRNA-binding capacity. Specifically, weak bands were observed at the height of the naked dsRNA control when complexes were formed at a 1:1_w:w_ or 2:1_w:w_, although partial complexation still occurred as evidenced by the light complex bands present in the wells (Figure 4, well 5 and 6). Full binding was achieved when HA2-penetratin and dsRNA were mixed at a ratio of 5:1_w:w_ (Figure 4, well 7). After a 5 min incubation in MgES, the bands representing the HA2-penetratin:dsRNA complexes immediately disappeared for all tested ratios, indicating that degradation took place almost immediately (Figure 4, well 9–12). The incubation experiment was therefore terminated at this timepoint.

HA2-TAT:dsRNA full complexation was achieved at a ratio of 2:1_w:w_, since the band at the height of the control dsRNA was no longer observed and complex bands were visualized in the well (Figure 5A–C, well 6). All complex bands in the wells disappeared after a 60 min incubation period, indicating that degradation occurred prior to this time point (Figure 5C, well 9–12). Because all tested complex ratios showed degradation after 60 min, the experiment was prematurely stopped at this point.

POA was saturated with ds*Tub* at a complexation ratio of 1:1_w:w_ (CPP:dsRNA), indicated by the complexation band formed in the well. While complexation was efficient for all tested ratios (Figure 6A–D, well 5–8), the 1:1_w:w_ and 2:1_w:w_ complex ratio bands showed an obvious decrease in intensity after a 5 min incubation period in MgES, indicative of degradation (Figure 6A, well 9 and 10). In fact, after 60 min, the bands in the well were no longer observed for these two complex ratios (Figure 6B, well 9 and 10). However, complexes formed at a 5:1_w:w_ and a 10:1_w:w_ ratio remained stable for the full duration of the experiment (Figure 6A–D, well 11 and 12).

Our results indicate EB1, C6M1, and POA as promising candidates to test for RNAi by dsRNA feeding in *S. gregaria*. We proceeded with EB1 for further in vivo investigation. Due to limited availability, POA and C6M1 were not further tested in this study.

### 3.2. Feeding EB1:dsRNA

**Figure 7:**
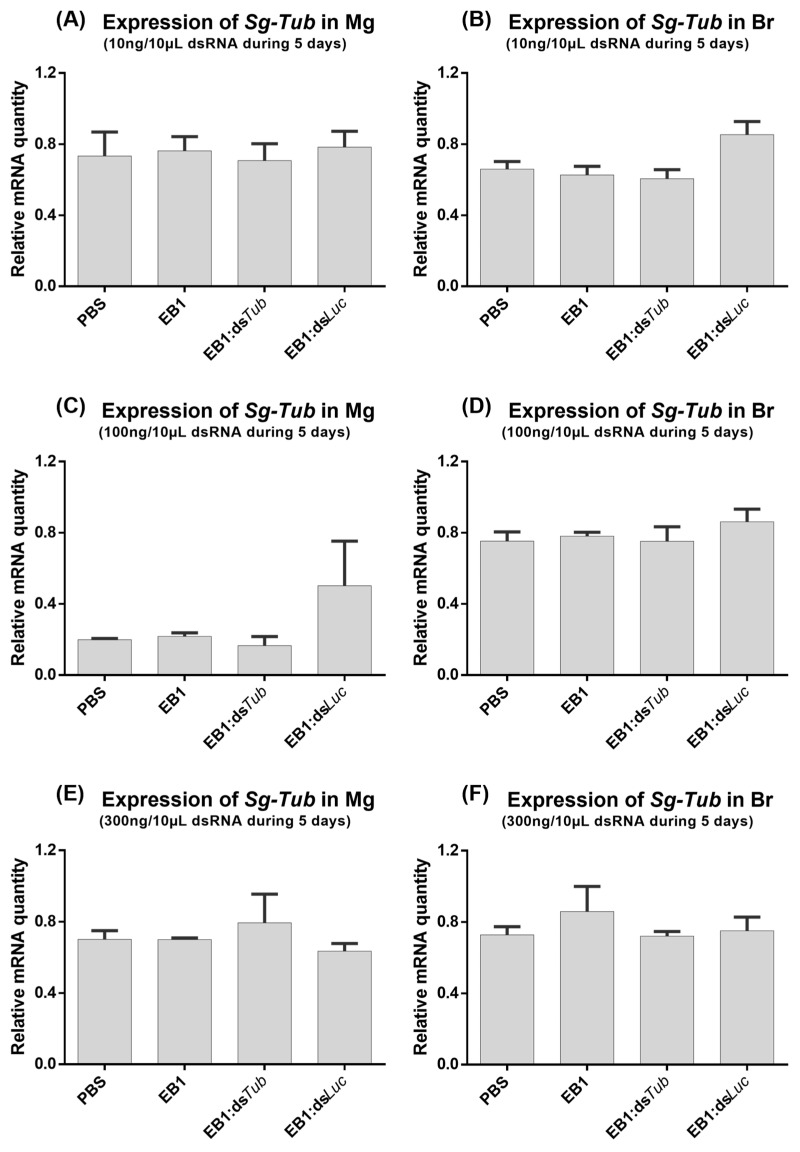
Relative expression of *tub* in the midgut (**A**,**C**,**E**) and brain (**B**,**D**,**F**) of *S. gregaria* after a 5-day feeding assay with 10 ng/10 μL (**A**,**B**), 100 ng/10 μL (**C**,**D**) and 300 ng/10 μL (**E**,**F**) EB1:ds*Tub* complex in a 5:1_w:w_ ratio. As a control, insects were similarly fed with PBS, EB1:ds*Luc*, or a comparative concentration of the naked peptide EB1. The bars represent the mean ± SEM of 3 independent pools of 3 insects, run in duplicate, and normalized against *GADPH* and *EF1α* levels for the midgut, or *EF1α* and *Ubi* for the brain. Abbreviations: *Tub: alpha-tubulin 1a*; Mg: midgut; Br: brain.

**Figure 8:**
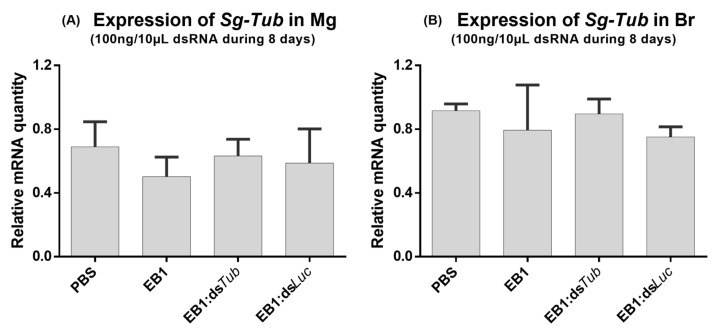
Relative expression of *tub* in the midgut (**A**) and brain (**B**) of *S. gregaria* after an 8-day feeding assay with 100 ng/10 μL EB1:ds*Tub* complex in a 5:1_w:w_ ratio. As a control, insects were similarly fed with PBS, EB1:*dsLuc*, or a comparative concentration of the naked peptide EB1. The bars represent the mean ± SEM of 3 independent pools of 3 insects, run in duplicate, and normalized against *GADPH* and *EF1α* levels for the midgut, or *EF1α* and *Ubi* for the brain. Abbreviations: *Tub: alpha-tubulin 1a*; Mg: midgut; Br: brain.

The ability of EB1:dsRNA complexes to induce RNAi-mediated gene silencing by feeding in *S. gregaria* was tested. For this, during five days, adult locusts were fed with 3 different quantities of dsRNA, and the quantity of peptide was always adjusted accordingly. The 5:1_w:w_ ratio was selected based on our previous screening (Section 3.1). EB1:dsRNA complexes were formed with ds*Tub* (marker gene dsRNA) or with ds*Luc* as a control (negative control). Furthermore, two extra control groups were analyzed in which the insects were fed with the naked peptide EB1 or with the solvent PBS. *Tub* was selected since its RNAi-mediated knockdown was previously reported to result in locust mortality [4]. The *Tub* transcript levels were then measured in the midgut (Mg) to assess if a local knockdown was induced, which would indicate that the dsRNA was successfully taken up from the midgut lumen. However, no transcript reductions were observed for any of the tested concentrations (Figure 7A,C,E). To test if the RNAi signal was transferred to other tissues of the insect body—systemic knockdown [2]—*Tub* levels were also measured in the brain. No downregulation of brain *Tub* transcript levels was observed (Figure 7B,D,F). Next, another feeding bioassay was performed but with one single dsRNA quantity and an extended feeding period of eight days. No transcript reductions were detected in either tissue (Figure 8).

### 3.3. Visualization of EB1:dsRNA and EB1:siRNA Complexes

**Figure 9:**
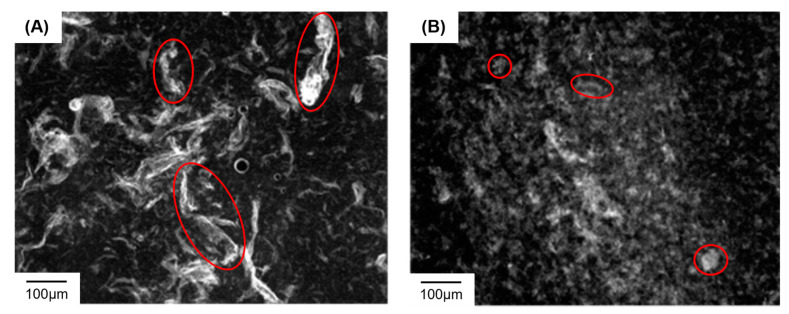
Stereoscopic images of EB1 complexed with (**A**) fluorescently labeled ds*Luc*, or (**B**) fluorescently labeled ds*Luc*-siRNA. Complexes were formed at a 5:1_w:w_ ratio (EB1:dsRNA). dsRNA or siRNA was labeled using the Cy3 fluorescent marker. Images were created with the AxioCam HRc camera (ZEISS) and complexed, fluorescently labeled dsRNA/siRNA was visualized using the SteREO discovery v8 microscope (ZEISS) at a wavelength of 532 nm and 8× magnification. Examples of complexes are indicated in red.

A requirement for any insect dsRNA delivery system is the ability to pass the alimentary barrier formed by the peritrophic membrane (PM). The PM compartmentalizes the insect digestive system, protecting gut epithelial cells from mechanical or chemical damage caused by ingested food [52]. Being a semi-permeable membrane, the PM contains pores, only allowing the uptake of food particles of a certain size [53]. Therefore, gut-mediated uptake of the EB1:dsRNA could be limited if these complexes exceed the size restriction implemented by the PM. In Orthoptera, this membrane is permeable to molecules with a diameter of 24–26 nm [54]. Thus, to gain insight into the size range of EB1:dsRNA, complexes were formed at a 5:1_w:w_ ratio with fluorescently labeled ds*Luc*, and visualized under a stereo microscope. We observed mainly large aggregates of >100 µm (Figure 9A), which are unlikely to pass the PM. In parallel, complexes were formed with fluorescently labeled *luciferase* siRNAs (ds*Luc*-siRNA). Although still possibly too large, complexation with ds*Luc*-siRNA noticeably resulted in a much smaller complex size (Figure 9B). We hence decided to further investigate EB1:siRNA complexes and their potential to induce RNAi by feeding.

### 3.4. EB1:siRNA Complexation and Stability in Ex Vivo Locust Midgut Environment

**Figure 10:**
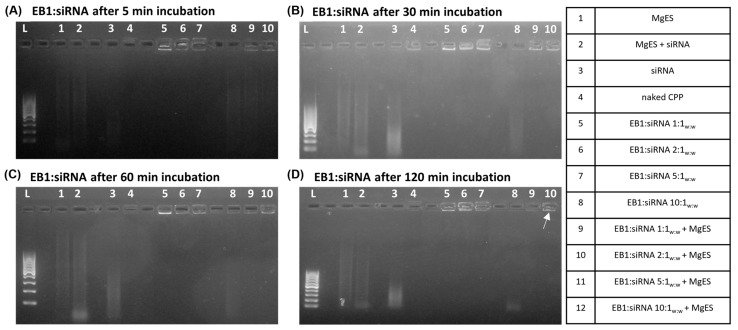
Ex vivo degradation assay of EB1:siRNA in MgES. Complexes were incubated for 5 min (**A**), 30 min (**B**), 60 min (**C**), and 120 min (**D**) to determine stability of EB1:siRNA in an *S. gregaria* midgut environment. A 200 bp ladder was used (L). As a control, incubated complexes were compared to pure MgES (1), naked siRNA incubated in MgES (2), naked siRNA (3), EB1 (4), and the native EB1:siRNA complexes at ratios of 1:1_w:w_ (5), 2:1_w:w_ (6), and 5:1_w:w_ (7). Complexes were incubated in MgES at ratios of 1:1_w:w_ (8), 2:1_w:w_ (9), and 5:1_w:w_ (10). A white arrow indicates the 5:1_w:w_ complex band after 120 min incubation in MgES. Furthermore, some background fluorescence was observed in all samples containing MgES.

EB1 was complexed with 50 ng/µL ds*Luc*-siRNA and incubated in MgES for periods of up to 2 h. The stability of the complexes in MgES incubation was measured at the time points 5 min, 30 min, 60 min, and 120 min. EB1:siRNA complexes were formed at 1:1_w:w_, 2:1_w:w_, and 5:1_w:w_. As a control, naked siRNAs were similarly incubated in MgES. Furthermore, native complexes, naked peptide, naked siRNA, and MgES, were also included as controls. All samples were analyzed through agarose gel electrophoresis (1%) and visualized using UV light. EB1 showed complete binding to ds*Luc*-siRNA at all tested ratios, forming complex bands in the well (Figure 10 A–D, well 5–7). When incubated in MgES, however, EB1:ds*Luc*-siRNA formed at a 1:1_w:w_ ratio quickly started to degrade after a 5 min incubation period, as evidenced by the reduced intensity of the band in the well (Figure 10A, well 8). While a clear band remained present for the complex formed at the 2:1_w:w_ ratio for at least 30 min (Figure 10B, well 9), band intensity appeared reduced after 60 min, indicating that the integrity of the complex was compromised (Figure 10C, well 9). Complexes formed at the 5:1_w:w_ ratio remained stable for the entire duration of the experiment, showing a clear band at the expected height even after 120 min of incubation in MgES (Figure 10D, well 10). This is indicated by a white arrow in the figure. As expected, naked siRNAs showed signs of further degradation after a 5 min incubation period (Figure 10A–D, well 2). Finally, MgES was visualized as a light smear of background fluorescence but no bands were observed (Figure 10A–D, well 1).

### 3.5. Injecting Naked siRNA

**Figure 11:**
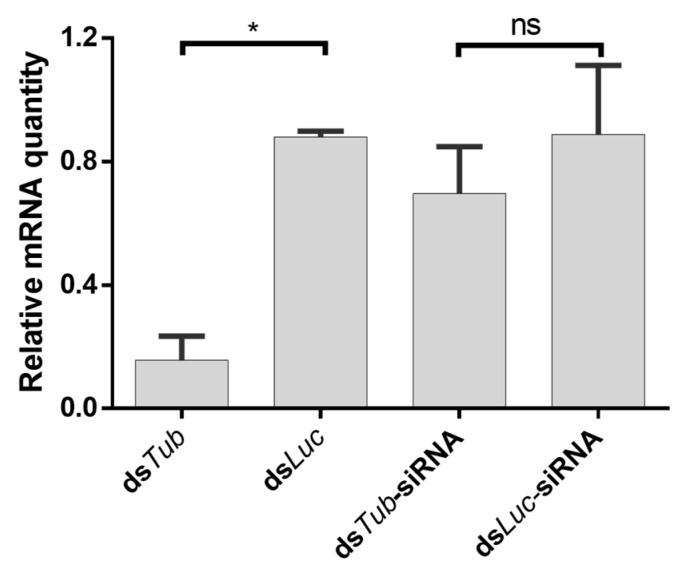
Relative expression of *tub* in the midgut of *S. gregaria* after injection with 500 ng of *siRNA dsTub*. Insects were treated with 500 ng/4 μL naked *dsTub*-siRNA, as well as 500 ng/4 μL ds*Luc*-siRNA. As positive and negative controls, ds*Tub* and ds*Luc* were tested. The bars represent the mean ± SEM of 3 independent pools (2 for siRNA ds*Luc*) of 3 insects, run in duplicate, and normalized against *GADPH* and *EF1α* levels. Data were analyzed using ANOVA (*p* = 0.0101), followed by Tukey’s multiple comparisons test (alpha = 0.05; * *p* < 0.05; ns: not significant). Abbreviations: *Tub: alpha-tubulin 1a*; Mg: midgut.

The uptake of naked dsRNA in insects is generally accepted to be length-dependent—it occurs efficiently for long dsRNA molecules, but not for shorter molecules, such as 18–24 nt siRNAs [55,56,57]. Nevertheless, siRNA-induced gene silencing has been reported in some species [58,59,60]. In this scope, we investigated if naked siRNAs trigger an RNAi response in locusts upon injection. For this, we injected locusts with ds*Tub*-siRNAs (marker gene) and ds*Luc*-siRNAs (negative control). In addition, groups injected with *Tub* or *Luc* long dsRNA were also included as a positive control. While injection of *Tub* dsRNA led to a potent knockdown, no downregulation was observed when ds*Tub*-siRNAs were injected (Figure 11).

### 3.6. Injecting EB1:siRNA

**Figure 12:**
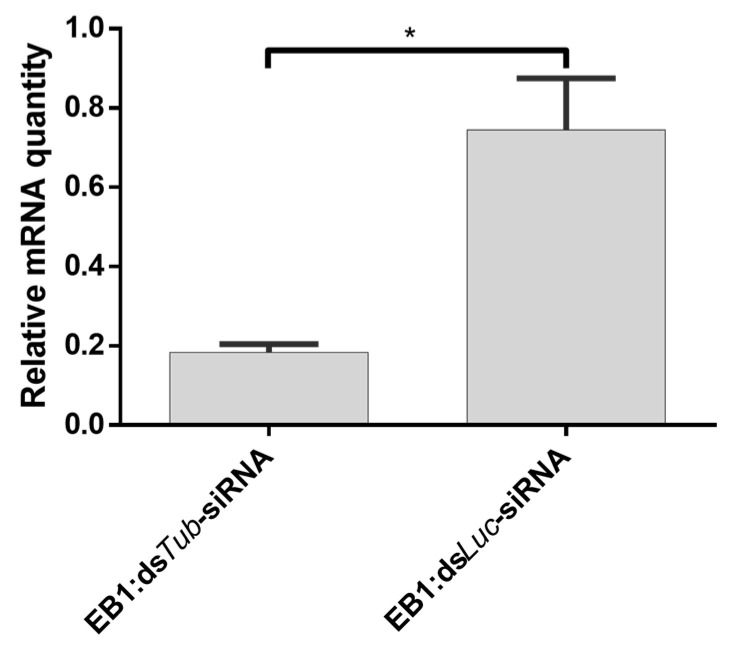
Relative expression of *tub* in the midgut of *S. gregaria* after injection with 500 ng/4 μL EB1:ds*Tub*-siRNA complex at a 5:1_w:w_ ratio. The knockdown was compared to EB1:ds*Luc*-siRNA. The bars represent the mean ± SEM of 3 independent pools of 3 insects, run in duplicate and normalized against *GADPH* and *EF1α* levels. Data were analyzed using unpaired *t* test (* *p* < 0.05); Abbreviations: *Tub: alpha-tubulin 1a*; Mg: midgut.

It was then assessed whether EB1:siRNA complexes could trigger RNAi in *S. gregaria* by injection. Insects injected with the EB1:ds*Tub*-siRNA complex showed a transcript reduction in comparison to insects treated with EB1:ds*Luc*-siRNA (Figure 12).

### 3.7. Feeding EB1:siRNA

**Figure 13:**
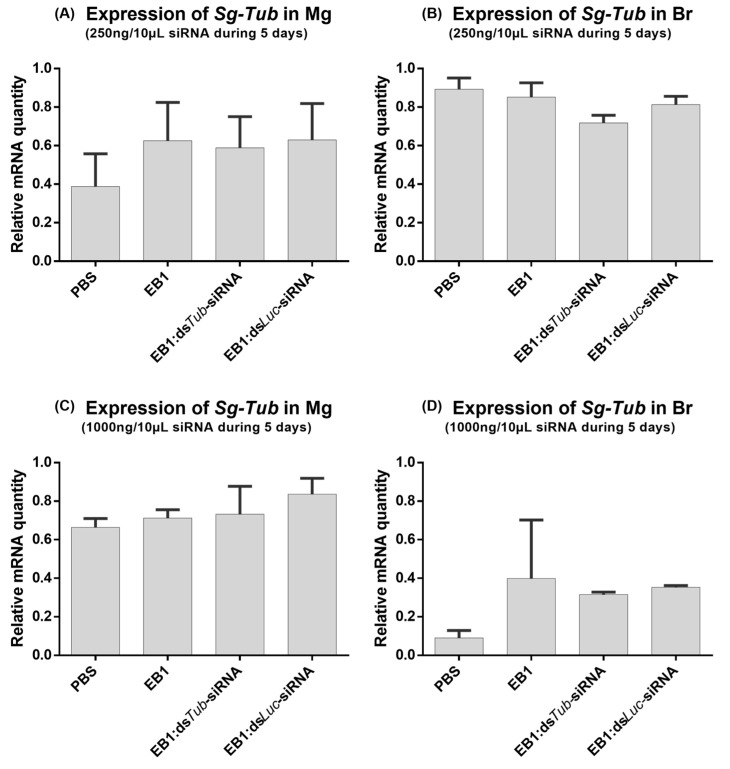
Relative expression of *tub* in the midgut (**A**,**C**) and brain (**B**,**D**) of *S. gregaria* after a 5-day feeding assay with 250 ng/10 μL (**A**,**B**) and 1000 ng/10 μL (**C**,**D**) EB1:ds*Tub*-siRNA complex in a 5:1_w:w_ ratio. As a control, insects were similarly fed with PBS, EB1:ds*Luc*-siRNA, or a comparative concentration of the naked peptide EB1. The bars represent the mean ± SEM of 3 independent pools of 3 insects, run in duplicate, and normalized against *GADPH* and *EF1α* levels for the midgut, or *EF1α* and *Ubi* for the brain. Abbreviations: *Tub: alpha-tubulin 1a*; Mg: midgut; Br: brain.

We then tested if EB1:siRNA complexes delivered by feeding could trigger an RNAi response in locusts. For this, a feeding bioassay in *S. gregaria* utilizing EB1 as an operative carrier for siRNA was performed. Complexes were formed at a 5:1_w:w_ ratio as initial screenings showed that these complexes could remain stable for up to two hours in an ex vivo *S. gregaria* midgut environment (Section 3.4). Thus, complexes were formed using 250 ng and 1000 ng ds*Tub*-siRNA or ds*Luc*-siRNA as a control, and dissolved in PBS. As an additional control, the effect of the solvent PBS and non-complexed EB1 on *Tub* expression was also tested. The quantity of EB1 (1250 ng or 5000 ng, respectively) was always adjusted to match the amount used to form the tested EB1:siRNA complex. Insects were force-fed with 10 µL of each condition for 5 days. *Tub* expression levels were measured both in the midgut to assess local knockdown and in the brain to assess systemic knockdown. Force-feeding of EB1:*dsTub*-siRNA at a concentration of 250 ng/10 μL per day, for five days, did not induce an RNAi response in either the brain or midgut of *S. gregaria* (Figure 13A,B). Likewise, increasing the concentration of the fed complex to 1000 ng/10 μL per day, for five days, did not induce any observable impact on *Tub* expression levels in both tested tissues (Figure 13C,D).

## 4. Discussion

A first step toward the global application of RNAi as an insecticide is to enable efficient silencing responses upon oral delivery of dsRNA. While some insects naturally respond to fed dsRNA, this is not the case in several species of interest. Two main limitations apply in this regard, namely (1) the degradation of the dsRNA prior to cellular uptake, due to the presence of dsRNA-degrading RNases in the digestive system; and (2) the absence of an efficient dsRNA-uptake mechanism in the gut. These problems can potentially be solved by the use of delivery systems, specifically designed to protect dsRNA from hostile gut environments and to facilitate local uptake.

The desert locust, *S. gregaria* is an agricultural pest of serious concern, which is constantly monitored by FAO (FAO Locust Watch: http://www.fao.org/ag/locusts accessed on 31 January 2023). While this species is very responsive to dsRNA delivered by intra-hemocoelic injection, it is completely refractory to orally delivered dsRNA [4,5]. In this study, five CPPs were tested for their potential to protect dsRNA in locust midgut, namely EB1, C6M1, HA2-penetratin, HA2-TAT, and POA. The design of this assay was based on the observation by Wynant et al. (2014) that enzyme solutions collected from the midgut of *S. gregaria* have a very potent nuclease activity, degrading dsRNA within 5 min. This activity can be traced back to the presence of four dsRNases in the locust gut [5]. Thus, incubating candidate CPP complexes in MgES provides a first indication of their ability to protect dsRNA against nuclease degradation.

We observed that the CPP penetratin derivative EB1 already showed complete binding to ds*Tub* at a 1:1 weight ratio (EB1:dsRNA). Moreover, as the ratio of peptide to dsRNA increased, so did the ability of the complex to withstand degradation in MgES (Figure 2). Thus, there is an apparent correlation between the amount of peptide and complex stability. This is in accordance with Xu et al. (2014) who showed that increased amounts of CPP improved complex stability [44]. Additionally, complex size often increases with the ratio of peptide to dsRNA [46]. As larger complexes will probably take more time to be fully digested, they will remain (partially) stable for a longer total period of time.

C6M1 achieved complete binding to ds*Tub* (545 bp) at a 5:1 weight ratio (C6M1:dsRNA). C6M1:dsRNA complexes showed remarkable stability in MgES, even when dsRNA was only partially complexed. In fact, dsRNA remained bound to C6M1 at all tested ratios and there were no clear signs of degradation (Figure 3). Interestingly, it has been previously reported that C6M1 protects small RNA duplexes from degradation in an active serum solution [46]. The stability of the C6M1:dsRNA complex may be inherent to the structure of the peptide as it was designed to adopt an α-helical amphipathic structure [44]. Concisely, this means that all positively charged arginine residues are concentrated on one side of the peptide, optimizing binding capacity and ultimately creating strongly associated dsRNA nanocomplexes.

Both HA2-penetratin and HA2-TAT showed low binding efficiencies, with HA2-penetratin achieving saturation at a weight ratio of 5:1 (HA2-penetratin:dsRNA) and HA2-TAT at 2:1 (HA2-TAT:dsRNA) (Figure 4 and Figure 5). Notably, HA2-penetratin and HA2-TAT contain the lowest amount of positively charged amino acids of all tested CPPs, i.e., both CPPs have a charge of 5+ in comparison to POA which has a charge of 12+ (Table 1). As the binding of ribonucleotides by the CPP is based on electrostatic interaction, the relative charge of the tested carrier peptide can have an important effect on its binding capacity for the negatively charged dsRNA. Thus, CPPs with a lower positive charge, such as HA2-TAT and HA2-penetratin, likely bind dsRNA less effectively, as is demonstrated here (Figure 4 and Figure 5). In addition, HA2-penetratin and HA2-TAT appeared unable to fully protect dsRNA under the tested conditions. While all complexes formed with HA2-penetratin were immediately degraded after a 5 min incubation period in MgES, HA2-TAT complexes remained stable for up to 60 min (Figure 4 and Figure 5). Notably, by using an in vitro gastric digestion mix, Chen and Li (2012) showed that peptides with a molecular weight (M_r_) of >3 kDa have a higher susceptibility to degradation than peptides with a molecular weight of <3 kDa [61]. Both HA2-penetratin and HA2-TAT have molecular weights > 3 kDa (Table 1), implying that they might be inherently less stable in gut environments.

Of all tested CPPs, POA appeared to have the strongest binding affinity (Figure 6). This is in line with a previous study by Unnamalai et al. (2004), which showed that a 0.5:1_w:w_ ratio (POA:dsRNA) was already sufficient to bind a 900 bp dsRNA construct [49]. This ability can mainly be attributed to the high amount of positive charges present in this peptide (Table 1). As the 1:1_w:w_ ratio was the lowest ratio tested here, it remains possible that POA binds dsRNA at even lower ratios, as indicated in the literature [49]. When these complexes were incubated in MgES, the 1:1_w:w_ and 1:2_w:w_ ratios immediately showed signs of degradation, with gel electrophoresis bands fully disappearing after 60 min. However, the 1:5_w:w_ and 1:10_w:w_ ratios remained stable for the full duration of the experiment, once again indicating a positive correlation between complex stability and complex ratio (Figure 2 and Figure 6).

Of note, both HA2-penetratin and HA2-TAT were created using naturally occurring sequences, while the other three CPPs were synthetically designed or modified. In fact, Lundberg et al. (2007) found that EB1 was more effective in binding siRNA at low ratios than its analog penetratin, indicating increased interaction with the nucleotide material [34]. The possibility therefore exists that the alterations implemented in these modified CPPs increase the general stability of the complex.

To our surprise, in vivo feeding assays with EB1:dsRNA resulted in no measurable knockdown (Figure 7 and Figure 8). We reasoned that this could be due to the large complex size (Figure 9A), which would impede its passage through the PM. The large EB1:dsRNA aggregates are likely created due to crosslinking, which often occurs when CPP complexes are formed through noncovalent linking [62,63]. Nevertheless, when the smaller EB1:siRNA complexes (Figure 9B) were tested by feeding, effective knockdown was still not observed (Figure 12). This is despite the fact that these complexes demonstrated stability in MgES (Figure 10) and that their injection was successful in triggering an RNAi response (Figure 11). Thus, it is still possible that the PM is acting as a barrier to the EB1:siRNA uptake—the complexes could still be inherently too large to pass the PM. In fact, orthopteran PM pores only allow passage of molecules with a 24–36 nm diameter range [54], and some of the observed EB1:siRNA complexes appear much larger than that (Figure 9B, red circles). More so, the complex size could also be affected by the utilized solvent. For instance, Jafari et al. (2014) found that C6M1:siRNA complexes dissolved in water had a size of approximately 70–155 nm. When these complexes were suspended in PBS, however, aggregation caused their size to increase to ~460 nm [46]. As PBS was also used as a solvent in our feeding assay, unforeseen aggregation could also contribute to creating complexes that are too big to pass through the pores of the PM.

On the other hand, it is interesting to speculate that although the dsRNA/siRNA are protected from degradation in the midgut when complexed with EB1, other steps of the digestive system could compromise the dsRNA integrity in the complex. For instance, dsRNA degradation activity has been shown in the saliva of insects such as *Lygus lineolaris* and *Nezara viridula* [64,65]. Moreover, even if dsRNA is not degraded, the lack of an efficient uptake system could pose a limiting factor. These ideas are in line with previous studies describing the lack of RNAi response upon dsRNA feeding in locusts, even when gut dsRNase activity was impaired [5,66].

Uptake of dsRNA into insect cells is reported to be length-dependent, occurring more efficiently for long dsRNA molecules, but not for shorter molecules such as 18–24 nt siRNAs [55,56,57]. However, response to siRNAs seems to vary among insect species, with some cases of success [58,59,60]. In this study, we show that, in *S. gregaria*, an intra-hemocoelic injection of siRNAs does not trigger an RNAi response, in contrast to an injection of long dsRNA (Figure 11). This is possibly due to an inefficient siRNA uptake, as described in other species [58,59,60]. Interestingly, intra-hemocoelic injection of siRNAs complexed with EB1 induces a silencing response (Figure 12). This is in line with the well-known capacity of some CPPs to perform intracellular delivery of nucleic acids [67].

Despite CPPs’ potential to facilitate the cellular uptake of a diverse range of molecules, including oligonucleotides, their use for insect-related applications remains underexplored. In this scope, we encourage further in vivo studies with C6M1, POA, and other CPPs, to explore RNAi by feeding in insects; as well as to explore their potential for the delivery of other oligonucleotide-based insecticidal approaches. Noteworthy, while laboratory in vitro synthesis of dsRNA/siRNA and of specific peptides might be costly, the tendency is for production costs to decrease once clear proof-of-concept has been delivered—for instance, due to further research efforts in efficient production methods and investment from specialized industry. This general trend is common among novel biotechnological products. In line with this, methods for dsRNA mass production with reduced costs are under investigation and several original approaches within the RNAi framework are being considered [17,68,69]. This emphasizes the potential of this gene-silencing pathway, as well as the importance of understanding its mechanisms.

In this study, we evaluated the capacity of five CPPs to bind long dsRNA, as well as to protect it from degradation by *S. gregaria* midgut enzymes. While EB1:dsRNA, C6M1:dsRNA, and POA:dsRNA complexes showed, to some extent, stability in MgES; both HA2-penetratin:dsRNA and HA2-TAT:dsRNA complexes were quickly degraded. We further investigated EB1:dsRNA complexes in vivo, which failed to induce RNAi by feeding in adult locusts. Similarly, although EB1:siRNA complexes remained stable in MgES, they also failed in triggering an RNAi response when fed to locusts. Interestingly, we observed that intra-hemocoelic injection of siRNA does not result in a silencing response in the locust, which can be overcome by the use of EB1:siRNA complexes. Our findings highlight the complexity of the dsRNA/siRNA-triggered RNAi in this locust species and emphasize the multifactorial nature of an efficient RNAi response in insects. In this context, it is of value to direct future research to insect- and tissue-specific uptake mechanisms of potential delivery systems. Moreover, our study emphasizes the importance of in vivo studies when it comes to dsRNA delivery systems.

## Figures and Tables

**Table 1:** CPP sequences, defining properties, origin, net charge at pH 7, and molecular weight (M_r_).

Name	Sequence	Properties	Origin	Charge (pH 7)	M_r_ (kDa)	Reference
EB1	LIRLWSHLIHIWFQNRRLK WKKK-amide	Endosomolytic, amphipatic.	Penetratin derivative.	+8	3.66	[34]
C6M1	RLWRLLWRLWRRLWRLLR	Endosomolytic, amphipatic.	Synthetic—based on C6.	+7	2.67	[44,45,46]
HA2-penetratin	GLFGAIAGFIENGWEGMI DGRQIKIWFQNRRMKW KK-amide	Fusogenic, amphipatic.	Chimeric—fusion of influenza virus hemagglutinin (HA) subunit and penetratin, derived from the third α-helix of the Drosophila Antennapedia homeodomain.	+5	4.28	[34]
HA2-TAT	GLFGAIAGFIENGWEGLIEG WYGGRKKRRQRRR	Fusogenic, amphipatic.	Chimeric—fusion of influenza virus hemagglutinin (HA) subunit and HIV-1 Trans-Activator of Transcription (TAT) protein.	+5	3.84	[47]
POA	RRRRRRRRRRRR	Cationic.	Synthetic—based on TAT peptide.	+12	1.89	[48,49]

**Table 2:** Oligonucleotide sequence of the primers used in the synthesis of *S. gregaria alpha-tubulin 1a* dsRNA [4]. The sequence in bold represents the T7 promoter site.

	Forward Primer	Reverse Primer
ds*Tub*	**taatacgactcactataggg** attttttagcgaaactggtgctggg	**taatacgactcactataggg** tggtgtaagtcgggcgttcaatgt

**Table 3:** Weight and molar ratios for CPP complexes formed with ds*Tub* (left; 545 bp, 341,000 g/mol) or ds*Luc*-siRNA (right; 18–26 bp, 13,300 g/mol). Molecular weights of the utilized CPPs can be found in Table 1.

CPP	Weight Ratio (CPP:dsRNA)	Molar Ratio (CPP:dsRNA)	Weight Ratio (CPP:siRNA)	Molar Ratio (CPP:siRNA)
EB1	1:1	93:1	1:1	4:1
	2:1	187:1	2:1	7:1
	5:1	466:1	5:1	18:1
	10:1	933:1		
C6M1	1:1	128:1		
	2:1	256:1		
	5:1	639:1		
	10:1	1278:1		
HA2-penetratin	1:1	80:1		
	2:1	159:1		
	5:1	399:1		
	10:1	797:1		
HA2-TAT	1:1	89:1		
	2:1	178:1		
	5:1	444:1		
	10:1	889:1		
POA	1:1	181:1		
	2:1	361:1		
	5:1	903:1		
	10:1	1806:1		

**Table 4:** Oligonucleotide sequence of the primers used in qRT-PCR, for the amplification of *alpha-tubulin 1a* (*Tub*), *glyceraldehyde phosphate dehydrogenase* (*GAPDH*), *elongation factor 1a* (*EF1a*) and *ubiquitin* (*Ubi*) [50].

	Fw Primer	Rv Primer
*Tub*	Tgacaatgaggccatctatg	cgcaaagatgctgtgattga
*GAPDH*	Gtctgatgacaacagtgcat	gtccatcacgccacacttc
*EF1a*	Gatgctccaggccacagaga	tgcacagtcggcctgtgat
*Ubi*	Gactttgaggtgtggcgtag	ggatcacaaacacagaacga

## Data Availability

All generated data are presented in Section 3 of this manuscript.
